# Investigating the relationship between stereotyping and creativity during marketing campaigns in marketeers and audiences

**DOI:** 10.1038/s41598-024-70704-z

**Published:** 2024-09-04

**Authors:** Nuoya Tan, Nimisha Parashar, Gorkan Ahmetoglu, Lasana T. Harris

**Affiliations:** 1 Present Address: Department of Management, University of Exeter, Exeter, UK; 2 Department of Clinical, Educational, and Health Psychology, University College London, London, UK; 3 Horizon Center for Doctoral Training, University of Nottingham, Nottingham, UK

**Keywords:** Stereotyping, Creativity, Advertising, Marketing, Human behaviour, Social behaviour

## Abstract

Stereotyping others in a creative process may negatively affect creative output, yet there is currently scant empirical evidence of a link between stereotyping and creativity; here, we explore this link in marketing communications. In a quasi-experiment, we introduced a novel intervention to disrupt marketeers’ dependency on stereotypes and boost their creativity. The intervention decreased marketeers’ use of stereotypes when selecting consumer labels—descriptive labels of a typical consumer based on consumer information—while enhancing the creativity of ideas. In another set of online experiments, we asked British residents to rate the creativity of advertisements and purchase intentions toward advertising products with different levels of stereotypical depictions of people. We found a linear relationship between the stereotypical depictions of people in advertisements and perceived creativity. We also observed a potential U-shaped relationship between stereotypical representations of people in advertisements and purchase intention, such that advertisements with low and high stereotypical representations induced greater purchase intention than did those with medium stereotypical representations. Finally, we discuss the psychological mechanisms that potentially link stereotyping and creativity and the implications for marketing communications.

Researchers have examined a possible link between social cognition and creativity^1^ that suggests both processes share similar psychological mechanisms. For example, priming stereotype-inconsistent information benefits creative thinking^2^, and priming a creative mindset encourages the use of stereotype-inconsistent information^3^. Inhibiting stereotyped responses relies on the executive function brain network^4,5^, parts of the brain that are also engaged during divergent thinking tasks^6,7^. This suggests that the act of regulating stereotyped responses may benefit creative endeavors. Given that stereotyping and creativity are two crucial concepts in marketing communications^8,9^, here we explore their possible link in advertising from the perspective of advertising creators and consumers. We are interested in marketeers’ use of stereotypes when using consumer information to select consumer labels that drive advertising creation and their creative thinking. To explore advertising consumption, we manipulate the stereotypical representation of people in advertisements and measure consumers’ perceived creativity of such advertisements.

## Stereotyping, creativity, and advertising

Flexible social cognition theory^10^ states that people hold multiple probable attributions for behavior when considering another person’s mental state. Some attributions are stereotypes—culturally held categorical information about another person’s perceived social group that provides information about that person’s traits and possible behaviors^11^. Other attributions are stereotype-irrelevant or category-inconsistent, such as traits inferred directly from behavior in a specified context. People can access either set of information based on their social goals or the social context. Stereotype avoidance occurs when people employ stereotype-irrelevant or stereotype-inconsistent information, which helps them perceive the person as unique^12^. Existing stereotype avoidance approaches include forming counter-stereotypical exemplars^13^, intentions not to stereotype^14^, rejecting stereotypical targets as valid^15^, and communicating a low level of stereotype consensus^16^.

Flexible social cognition theory^10^ can be considered a special (social) case of flexibility mindsets theory^3^. The flexibility mindsets theory argues that people have dominant and spontaneous response inclinations (associations) when they process information (e.g. about people or objects) and if the dominant response is broken (e.g. social expectation violation), people can become more flexible, or divergent, in their thinking. Since information processing is not tied to a specific situation, flexible thinking can carry over from one situation to another. That is, breaking a dominant response to people, may trigger nondominant response options being considered for objects and vice versa^17^. Thus, creativity may rely on similar processes to stereotype avoidance, since both processes involve making associations between nondominant and remotely related concepts.

Consistent with this view, previous research has shown that activating stereotype-inconsistent information in one’s mind helps generate creative ideas. For example, people were more cognitively flexible (operationalized as a measure of creative thinking) in a pasta-naming task when they were asked to generate words to describe a female (stereotype-inconsistent) rather than a male mechanic (stereotype-consistent)^18^. Priming multiracial identities benefited multiracial people’s convergent and monoracial people’s divergent thinking while priming a mono-racial mindset did not^18^. People designed a more creative poster for a nightclub when required to generate stereotype-inconsistent rather than stereotype-consistent category combinations^2,19^. Stereotype-inconsistent information led to more creative responses on a Chinese idiom riddle test^20^. Moreover, priming a creative mindset reduced stereotype activation; people showed slower responses in a lexical decision task toward stereotypical targets after they wrote about creative ideas than after they wrote about thoughtful ideas or wrote nothing^21^.

Why does this research matter for advertising? Audiences positively react to advertising creativity^22^. Compared with noncreative advertisements, creative advertisements elicit deeper information processing^23^ and a higher level of perceived product quality^24^. In addition, audiences exhibit greater attention^25^, liking, memory recall^26^, and purchase intentions^27^ toward creative advertisements and corresponding brands and products. However, advertisements tend to rely on stereotypes, including overrepresenting ideal imagery (e.g., skinny women)^28^, underrepresenting minority groups (e.g., homosexual couples)^29^, and representing some social groups with generalized biases (e.g., older people depicted with poor health or stay-at-home spouses as undereducated)^30^.

The convenience of communication might be a reason for advertising stereotypes. In advertising creation, stereotypes may require fewer cognitive resources for marketeers to generate as they are congruent with widespread societal beliefs about social reality^31^. In advertising consumption, processing stereotypes might save the audience’s cognitive resources to digest sales stimuli in the advertisements^32^. Certain stereotyped imagery might trigger audience’s social comparisons^33,34^, which may drive their purchase intentions^35^. The potential benefit to sales may motivate marketeers to deliver stereotypical imagery to audience s.

Nonetheless, the stereotyped imagery, either positive or negative, may have negative societal impacts. For example, exposuring to positive stereotypes toward African Americans was detrimental to participants’ egalitarian social perception and increased their application of prejudicial beliefs^36^. When stereotypes were negative, they significantly undermined the cognitive performance and well-being of the stigmatized individuals^37^.

Audiences also react negatively toward stereotypes in advertising. For example, stereotypical information about females (e.g., physical characteristics and occupational roles) induced negative responses (e.g., negative arousal and defensive responses) that led to audiences’ negative attitudes toward the advertisement and the advertised brand^9^. In addition, audiences reacted negatively when advertisements attempt to persuade them by using stereotypes^38^. Members of minority groups showed negative attitudes toward stereotypical advertisements and brands and low purchase intentions toward the products^39^. Furthermore, minority group stereotypes elicited negative reactions in majority groups^40^.

Conversely, advertisements that avoid stereotypes elicit positive audience responses. For example, representing minority groups in advertisements induced a mismatch between audiences’ expectations and perceived information^41^, which motivated in-depth information processing of advertising products^42^. For people who were open-minded toward minority groups, in-depth information processing induced self-categorization change^43^, increased social connectedness and empathy and evoked positive reactions toward the corresponding social groups and advertising brands^44^. People with negative impressions of minority groups perceived the advertisement as irrelevant and reacted neutrally^45^.

Therefore, despite the limits of priming approaches popular in the above studies, stereotypical displays of people in advertising may have both positive and negative impacts, potentially driving purchase behavior and sales, but harming brand reputation and society more generally. This may explain why brands are increasingly moving away from stereotyped images of people in their advertising and the change in norms and regulations around stereotypes in advertising more generally^46^.

Here, we further link stereotyping and creativity in advertising. In the initial study, we test whether a stereotype intervention would cause real-world marketeers to think more creatively about their ordinary consumers. We predict that the stereotype intervention reduces marketeers’ stereotypical thinking about a persona from consumer information and benefits their creative thinking. In the second study, we focus on audiences and test whether advertisements of varying degrees of stereotypical representations of people would be evaluated differently on advertising creativity and lead to different purchase intentions for the advertised product or service. We predict that stereotypical representations of people in advertisements are associated with audiences’ perceived creativity of advertisements and purchase intention toward advertising products.

## Study 1a

### Participants

Using Amazon Mechanical Turk (Mturk), we randomly recruited participants (*N* = 152, 69 women, *M*_age_ = 33.30 years, *SD*_age_ = 9.73) who resided in the USA (68%), the UK (29%), or the Netherlands because the brand team we worked with for this study were resided in these three countries. Countries that are geographically and culturally close to the three countries, such as Canada were also included (3%) since they were likely to share similar stereotypes with our marketeers (study 1b participants). We recruited participants based on a first-come-first-served principle. They gave informed consent and received £7.50 / hour. The sample size fulfilled a predetermined project budget. Post-hoc power analyses via GPower suggested that we had enough power (0.99) to detect the chi-square effect on consumer label selection frequency at the *p* = 0.05 level (effect size *w* = 0.50).

### Materials

#### CLT

We began with a pilot study to develop a consumer labeling task (CLT) that benchmarked consumer labels’ stereotypicality as a measure of marketeers’ stereotypical thinking. The CLT reflected a crucial decision point for marketeers in the process of real-world media communications including advertising design, in which they were asked to indicate associations of consumer persona with consumer labels.

The *consumer persona* represents key information of a segment of an audience, such as attitudes toward life (e.g., “life is too boring, needs more adventure and excitement”), brand preference (e.g., buyer index for cable TV networks), hobbies (e.g., playing sports, reading), and gender split (e.g., male (42%) and female (58%)). The *consumer labels* are keywords, such as creative and eco-friendly, that may be used to characterize the persona. To create a real-world context, we neither clarified nor explained consumer labels to participants. In other words, we cared about how participants label others in real life, where labels had subjective meanings to individuals. All consumer data and consumer persona labels came from actual information provided by the participating brands.

We asked participants to read real-world consumer persona and select ‘many’, ‘few’, or ‘none’ amongst seventeen consumer labels to characterize the given persona. The participants ranked the selected labels based on how much they would like to use the label to characterize the persona. The higher the ranking of a label was, the more likely the participants were to use the label to characterize the persona.

We summed the frequency (Eq. (1)) that each consumer label was selected (selection frequency of labels).

1 $${\text{F}}_{{{\text{CL}}}} = \left( {\left( {\sum {\left( {{\text{S}}_{{{\text{CL}}}} } \right)} } \right)/{\text{N}}_{{{\text{CL}}}} } \right)* \, 100$$

where F_CL_ represents consumer label frequency, S_CL_ represents the selection of a label across all participants, and N_CL_ represents the number of all selections.

The more frequently participants selected a consumer label for a consumer persona, the more stereotypical the consumer label was toward the consumer persona. The consumer label stereotypicality (equation 2) was the average reverse score of all rankings for the consumer label. ST_CL_

2 $${\text{ST}}_{{{\text{CL}}}} = \frac{{\mathop \sum \nolimits_{{\text{i = 1}}}^{{\text{N}}} 18{ } - {\text{ ri }}}}{{\text{N}}},{\text{r }} \in \left( {1,18} \right)$$

where ST_CL_ represents consumer label stereotypicality, *r* represents the label rank of participants; and for non-selected labels, *r* = 18. The more frequently participants selected a consumer label or the higher the label ranking was, the more stereotypical the consumer label was toward the consumer persona. We then averaged consumer labels’ selection frequency and stereotypicality across participants. The participants’ stereotypical thinking equaled the sum of the stereotypicality of their selected labels.

### Procedure

Participants completed two CLTs with different consumer personas but the same consumer labels. Consumer labels were presented in random order. The participants also described the personas in open-ended questions—we do not discuss these qualitative data further since it deviates from our research focus.

## Results

### Consumer labels

We report the selection frequency, stereotypicality, and ranking of consumer labels to two consumer personas in Table 1. We conducted a chi-square analysis to determine whether the selection of consumer labels significantly differed. We also conducted a paired sample *t*-test to determine whether the stereotypicality of the labels at higher stereotypical ranking positions was significantly greater than that at lower stereotypical ranking positions.

**Table 1:** Descriptive Statistics of Consumer Labels in CLTs in Study 1a.

Consumer labels	CLT 1			CLT 2		
	Freq. (%)	Stereo	Rank	Freq. (%)	Stereo	Rank
Creative	6.09	5.24	7	9.64	7.15	2
Eco-friendly	4.79	3.82	11	5.38	3.74	9
Risk seeker	4.46	3.44	13	5.88	3.97	8
Active	7.40	6.33	3	13.02	10.00	1
Loyal	5.22	4.27	10	6.88	5.27	7
Unhealthy	9.03	6.75	2	3.88	2.16	13
Price driven	6.53	5.14	8	4.38	2.67	10
Anxious	7.29	5.78	4	3.00	1.68	14
Adventurous	5.99	4.63	9	9.26	6.72	3
Lazy	6.86	5.55	5	2.75	1.58	15
Security preferred	4.46	3.35	14	3.88	2.60	11
Convenience preferred	8.92	7.54	1	3.50	2.21	12
Self-conscious	6.86	5.44	6	7.13	5.37	6
Socializing	4.46	3.31	15	7.89	5.75	5
Status driven	4.68	3.52	12	8.51	6.37	4
Fickle	3.81	2.79	16	2.50	1.36	17
Introverted	3.16	2.12	17	2.50	1.54	16

Freq. refers to frequency. Stereo. refers to consumer label stereotypicality. Rank. refers to ranking of labels based on stereo. (from the most to the least).

For example, in task 1, the proportion of participants who selected the most stereotypical label *convenience preferred* did not differ from those who selected *active*, *creative*, *adventurous*, *loyal*, *eco-friendly*, and *socializing* labels, and significantly differed from those who selected the other ten labels. The stereotypicality of the most stereotypical label *convenience preferred* was not significantly greater than that of the second and the third stereotypical labels (i.e., *unhealthy* and *active*) and was significantly greater than that of the other thirteen labels. The chi-square and pair sample t statistics of each pair of consumer labels for the two tasks can be found in Supplementary Tables S1–S5 online.

## Study 1b

Armed with the CLT, we next considered the link between stereotyping and creative thinking in the labeling stage of the generation of the advertisement. We employed a 2 *condition* (intervention vs. control) X 2 *time* (pre-intervention vs. post-intervention) mixed design, with the former as a between-subjects factor. We measured whether stereotype intervention impacted marketeers’ stereotypicality in CLT and creativity in the Alternative Uses Task (AUT)^47^.

### Participants

We recruited marketeers using convenient samples and snowballing techniques. All marketeers in a multinational company received workshop invitations (the stereotype intervention was promoted to marketeers as a workshop to boost creativity, not as a stereotype intervention). The marketeers who accepted and joined the workshop were in the intervention group, and the others were in the control group.

One hundred thirty-six marketeers joined the study voluntarily, whereas only 47.1% completed the main tasks (*N* = 53, 33 women, *M*_age_ = 36.38 years, *SD*_age_ = 8.42), which constituted valid responses. Post-hoc power analyses using GPower suggested that we had enough power (1.00) to detect the *condition* × *time* interaction effect on creativity at the *p* = 0.05 level (partial *η*^2^ = 0.09, effect size* f* = 0.31). Thirty-four participants were in the intervention group, and 19 were in the control group, working in the UK (55%), the USA (26%), the Netherlands (13%), or other European countries (6%).

### Materials

#### CLT

We used CLTs to measure participants’ stereotypicality at the crucial decision point in advertisement development. Please see study 1a for details.

#### AUT

We measured creativity in AUT where participants thought of as many uses as possible for a brick and a mug. We assigned an originality score to each use based on the AUT frequency (Eq. (3)), such that a generic sample produced an answer.

3 $$AUT\; frequency = \frac{Occurance \,of \;a\; use\; across\; all\; participants}{{The\; number\; of\; all\; uses}} \times 100$$

If the frequency of an answer was 5% or above, the originality score was 0. If the frequency of an answer was between 1 and 5%, the originality was 1. If the frequency was less than 1%, the originality score was 2. We averaged the originality scores of the uses across the generic sample and calculated marketeers’ AUT originality (Eq. (4)).

4 $$AUT \;originalty = \frac{Average\;originality\;score\;of\;uses}{Fluency}$$

in which fluency is the number of appropriate uses. The greater the originality and fluency were, the greater the level of creativity.

### Procedure

Participants provided informed consent and completed an online pre-intervention test consisting of two AUTs, a CLT, a gender bias scale, and demographic information. The intervention group was exposed to the stereotype intervention the following day, whereas the control group did not receive any deliberate intervention.

There were two parts to the intervention. In the first part, participants completed a deoxyribonucleic acid (DNA) swab and received ancestral DNA information along with their team members who were also participants. They then joined a brief lecture that provided a primer on DNA and information on how to interpret their results. This aspect of the study was included for promotional purposes and will not be discussed further.

In the second part, usually the following day, we focused on stereotype reduction in a one-day workshop. This approach made salient the marketeers’ professional selves and their responsibility to the public—to be creative and to avoid perpetuating negative stereotypes. Building on their DNA experience, we discussed psychological illusions that suggested perception could differ from reality. We linked stereotypes and creativity, explaining that moving away from reliance on stereotypes could boost the creativity of advertisements. We discussed how stereotypes are acquired and processed in the brain. We gave them opportunities to discuss within their brand teams what processes may better avoid reliance on contextual events that promote the use of stereotypes as heuristics. We provided them with a toolkit to combat the employment of stereotype-consistent information. We also asked participants to reflect on their creative thinking process and identify the pressure points where stereotype activation was likely to occur, for example, deadlines that promote heuristic thinking.

The intervention group completed a post-intervention test two weeks after the intervention, and the control group completed a post-intervention test three days after the pre-intervention test. The post-intervention test repeated all tasks in the pre-intervention test plus a novel CLT that examined participants’ stereotypicality toward a new consumer persona.

## Results

### Statistical assumptions

To prepare an appropriate dataset for mixed repeated measures ANOVA, we performed a series of assumption tests on the variables. All the variables met the assumptions of nonzero variances, independence of variables, homogeneity of variance, and sphericity. There were no outliers. The skewness (between ± 2) and kurtosis (between ± 2) indicated that the data contained approximately normally distributed error. Descriptive statistics and normality tests of all the measures can be found in Supplementary Tables S6–S7 online.

### Stereotypicality

To examine the effect of the stereotype intervention on marketeers’ stereotypical thinking, we performed a mixed measures ANOVA on CLT stereotypicality. There was a significant main effect of *time*, *F* (1, 51) = 23.15, *p* < 0.001, partial *η*^2^ = 0.31, *Ω* = 0.67, such that stereotypicality before the intervention (*M* = 794.23, *SD* = 303.09) was greater than that after the intervention (*M* = 591.00, *SD* = 290.55).

The *condition* × *time* interaction did not have a significant effect. We then conducted simple effect tests to probe our hypothesis that the stereotype intervention reduces marketeers’ stereotypical thinking about a persona from consumer information. There was a significant difference between the intervention and control groups after the stereotype intervention, *t* (51) =  − 2.07, *p* = 0.044, 95% CI [− 167.00, − 80.72], such that stereotypicality was lower for the intervention group (*M* = 531.13, *SD* = 315.35) than for the control group (*M* = 698.13, *SD* = 206.59). There were no significant differences in stereotypicality between the intervention and control groups before the intervention.

There was also a significant difference between stereotypicality in the intervention group, *t* (33) = 4.96, *p* < 0.001, 95% CI [144.30, 345.26], such that stereotypicality before the intervention (*M* = 775.91, *SD* = 308.79) was greater than that after the intervention (*M* = 531.13, *SD* = 315.35). There was also a significant difference between stereotypicality in the control group, *t* (18) = 2.37, *p* = 0.029, 95% CI [14.59, 243.14], such that stereotypicality was greater before the intervention (*M* = 827.00, *SD* = 298.01) than after the intervention (*M* = 698.13, *SD* = 206.59).

Following the significant differences pre- to post-intervention in both the intervention and control groups, we compared pre-intervention stereotypicality in the CLT (task 1) with post-intervention stereotypicality in the repeated CLT (task 1) and the novel CLT (task 2), respectively. In the intervention group, there was a significant reduction in stereotypicality in the repeated CLT, *t* (33) = 4.19, *p* < 0.001, 95% CI [134.13, 386.81], and the novel CLT, *t* (33) = 3.64, *p* < 0.001, 95% CI [101.20, 356.98], such that stereotypicality was greater in pre-intervention CLT than the repeated CLT (*M* = 515.44, *SD* = 375.73) and the novel CLT (*M* = 546.82, *SD* = 396.65) in post-intervention.

In the control group, there was a significant reduction in stereotypicality in the repeated CLT, *t* (18) = 3.40, *p* = 0.003, 95% CI [64.61, 274.13], such that stereotypicality was greater in pre-intervention than in post-intervention (*M* = 657.63, *SD* = 182.99). However, there was no significant reduction in stereotypicality in the novel CLT. The different novel CLT performances between the two groups implied that our intervention decreased stereotypicality in the intervention group for both repeated and novel CLTs, whereas the time lapse decreased stereotypicality in the control group for repeated CLT only.

### Creativity

#### Originality

**Fig. 1:**
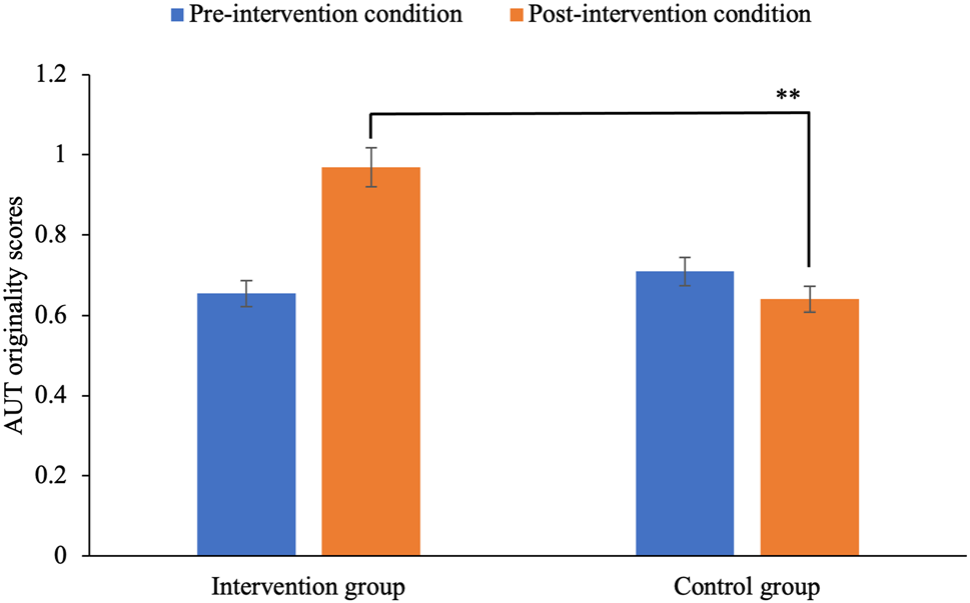
Originality scores in the Alternative Uses Task (AUT) (with 95% confidence intervals) of intervention and control groups in pre-intervention and post-intervention conditions in study 1b. AUT originality score that shows a significant effect of stereotype intervention is marked with two asterisks (*p* < .01); error bars represents ± 1 SE are shown.

To examine the effect of stereotype intervention on creativity, we performed a mixed ANOVA on AUT originality (please see Study 1b Materials for calculation and details). Neither of the main effects revealed significant differences. However, there was a significant *condition* × *time* interaction, *F* (1, 51) = 5.22, *p* = 0.027, partial *η*^2^ = 0.09, *Ω* = 0.31 (see Fig. 1). We followed up this interaction with simple effect tests. There was a significant difference between originality in the intervention group, *t* (33) =  − 2.82, *p* = 0.008, 95% CI [− 0.54, − 0.09], such that originality was lower in pre-intervention (*M* = 0.65, SD = 0.32) than post-intervention (*M* = 0.97, *SD* = 0.72). Originality in the control group did not reveal significant differences between pre-intervention (*M* = 0.70, *SD* = 0.52) to post-intervention (*M* = 0.64, *SD* = 0.52).

#### Fluency

We performed a mixed ANOVA on AUT fluency to determine whether the marketeers produced more or fewer ideas after the intervention, which also represented their creativity level (please see Study 1b Materials for calculation and details). Neither the main effect nor the interaction revealed significant differences.

### Exploratory analysis

According to the above results, the effects of the stereotype intervention on CLT stereotypicality and AUT originality followed the same pattern. Therefore, we conducted mediation analyses to examine whether the significant effects on CLT stereotypicality were mediated by AUT originality. We examined whether the significant effect of the *intervention* on post-intervention stereotypicality was mediated by post-intervention originality. The indirect effects revealed that the relationship between post-intervention stereotypicality and post-intervention originality was insignificant. We examined whether the significant effect of *time* on stereotypicality was mediated by originality in the intervention group. The indirect effects showed that the relationship between stereotypicality and originality in the intervention group was insignificant. The results implied that AUT originality did not mediate the effect of the *intervention* on CLT stereotypicality.

## Discussion

The results of study 1 support our prediction and show that the intervention, which encouraged stereotype avoidance, decreased marketeers’ stereotypical inferences, and improved their ability to produce original ideas. This finding is consistent with flexibility mindsets theory, which indicates that breaking dominant associations about people (i.e., stereotypes) can trigger flexible and divergent ideas (i.e. creative thinking) more generally, including about the uses of objects.

Importantly, previous studies have validated a causal effect of stereotypes on creativity. Since we activated both stereotype avoidance and creativity in the intervention, we could not draw such a causal relationship conclusion from our findings. We also note that it is possible that the DNA component had an impact (positive or negative) on the overall intervention, but we cannot determine such effects in the current study; thus, this is an open question for future research.

## Study 2a

Study 1 investigated the link between stereotype avoidance and creative thinking from the perspective of advertising generation. We next look at the link from the perspective of advertising consumption. To create appropriate stimuli for the main study (study 2b), we conducted a pilot study that measured audiences’ perception of groups with protected characteristics in a series of video advertisements.

### Participants

Using Prolific Academic, we randomly recruited participants (*N* = 61, women = 39, *M*_age_ = 31.60 years, *SD*_age_ = 10.68), who were residents in the UK for more than five years, based on a first-come-first-served principle. The 5-year UK residence was intended to ensure that our sample was aware of British societal stereotypes. Post-hoc power analyses using GPower suggested that we had enough power (1.00) to detect the association between familiarity and positive emotion at the *p* = 0.05 level (coefficient of confirmation *r*^*2*^ = 0.47, effect size *|r|*= 0.69).

Participants consisted of White British (85%), Asian British (7%), Black British (5%), and mixed ethnicities (3%). More than half of the participants had a bachelor’s degree (66%) and did not report a long-standing disability (92%). They reported themselves as heterosexual (75%), LGBTQ (21%), or preferred not to say (4%). The participants gave informed consent and received £7.50/hour.

### Materials

#### Advertisement pool

We procured 66 videos from an online database (https://adsoftheworld.com) that contained advertisements aired in the UK. These 66 video advertisements satisfied three criteria. First, the advertisements were disseminated in 2017, three years before data collection, so they were neither too old to be irrelevant nor too recent to be highly memorable for UK residents. Second, the length of each advertisement was 30 s to facilitate sufficient stimuli to boost statistical power without placing an undue burden on participants. Third, there were all live-action videos featuring human actors only instead of animation or animated objects or animals. We excluded the animated videos because most of the advertisements in the database were live-action videos featuring human actors, and only a few of them were animated ones. Therefore, excluding animated stimuli could ensure consistency in the presented stimuli without biasing the selection of the materials. Advertisement video examples are available upon request.

#### Familiarity

We measured familiarity with each advertisement. Participants responded to “How familiar are you with the advertisement?” on a 7-point Likert scale. The higher the rating was, the greater the familiarity with the advertisement.

#### Perceived stereotypicality

We measured perceived stereotypicality toward each advertisement with four question sets. Each question set started with a yes–no question to evaluate whether an advertisement represented one protected characteristic social group (i.e., women, LGBTQIA + , BAME, or disabled individuals). If yes, participants assessed the degree of representativeness, significance, stereotypicality, and social interactions for the social group on a 7-point Likert scale. As shown in the advertisements, “How explicit is the character to its social category (e.g., women)?” “How significant a role did the character play in the advertisement?” “How stereotypical is the role being performed by the character?” and “How positive/negative would you describe the interaction between the character in the social group and the characters from other groups?". If no, we proceeded the participants to the next question set. After answering four question sets about the four social groups, the participants rated the advertisement’s overall stereotypicality on a 7-point Likert scale. The perceived stereotypicality of an advertisement equaled the sum of representativeness, significance, interaction, and inclusivity ratings minus the stereotypicality rating (Cronbach’s *α* = 0.60). The higher the calculated ratings were, the less stereotypical the advertisement.

#### Perceived creativity

We measured perceived creativity toward each advertisement using a validated instrument^48^. The participants rated how much they agreed/disagreed with five statements on a 7-point Likert scale: “The advertisement was different”. “The advertisement was uncommon”. “The advertisement was relevant to you”. “The advertisement was meaningful to you”. and “How creative do you think the advertisement was?”. We summed the ratings of the five statements (Cronbach’s *α* = 0.94). The higher the summed ratings were, the greater the creativity the participants perceived from the advertisement.

#### Emotion valence

To measure the level of positive and negative emotions elicited by each advertisement, participants rated emotional valence with two statements on a 7-point Likert scale. “How positive does the advertisement make you feel?” and “How negatively does the advertisement make you feel?”^8^. The higher the rating was, the higher the level of the rated emotion.

### Procedure

In the beginning, all participants gave consent and reported the length of their UK residence. We proceeded only with those living in the UK for five years or more. There were 22 rounds of advertisement evaluations with a time limit of 5 min per round. In each round, the participants watched an advertisement and rated on familiarity, perceived stereotypicality, perceived creativity, and emotional valence. The advertisements were randomly selected from our advertisements pool and did not repeat. The participants then provided their demographic information.

## Results

### Statistical assumption

To prepare an appropriate dataset for Pearson correlations, we tested relevant assumptions for such tests. All the variables met the assumptions of related pairs, linearity, and homogeneity of variance. There was no outlier. The skewness (between ± 3) and kurtosis (between ± 5) indicated that the data contained approximately normally distributed error. See descriptive statistics and normality tests of all measures in Supplementary Tables S8–S9 online.

### Audiences' perception

**Fig. 2:**
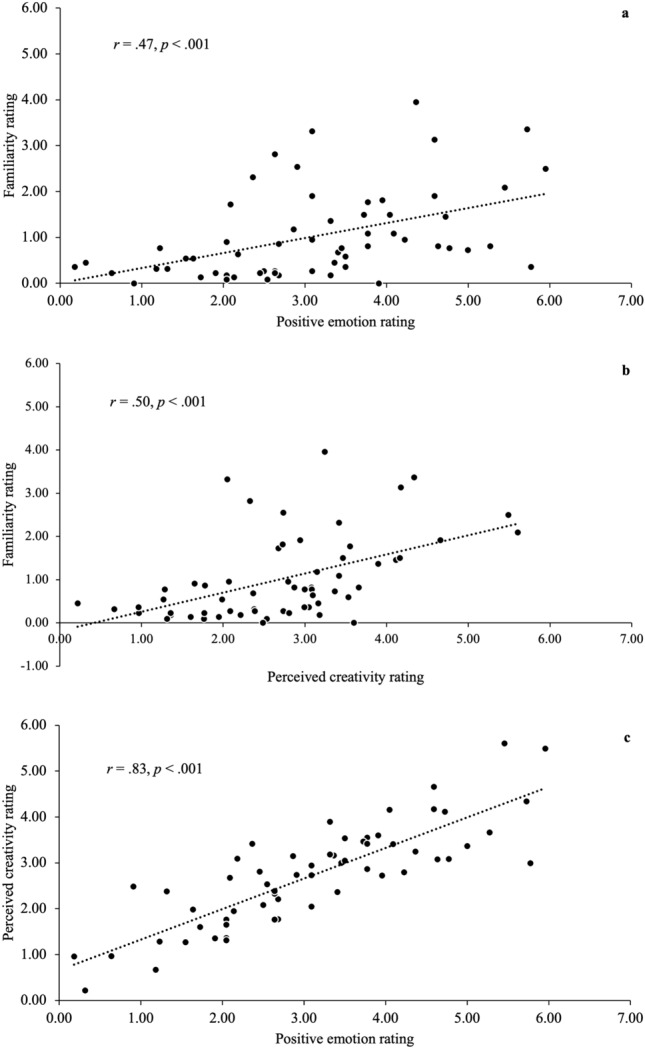
Associations among the familiarity, positive emotion, and perceived creativity toward advertisements in study 2a. Best fit lines with r values are shown.

Pearson correlation results indicated that familiarity, positive emotion, and perceived creativity toward advertisements yielded significant positive associations (see Fig. 2 and Table 2). Perceived stereotypicality and negative emotion were associated with neither of the other variables.

**Table 2:** Descriptive Statisctics of Familiarity, Perceived Stereotypicality, Perceived Creativity, Postive Emotion, and Negative Emotion and Their Correlations in study 2a*.*

Variable	M	SD	1	2	3	4	5
1. Familiarity	1.02	0.96	–				
2. Perceived stereotypicality	11.08	4.67	− .12	–			
3. Perceived creativity	2.72	1.09	.50**	.13	–		
4. Positive emotion	3.09	1.37	.47**	.20	.83**	–	
5. Negative emotion	1.18	0.88	.04	− .07	.12	− .02	–

*M* = mean; *SD* = standard deviation.

**p < .01 for *N* = 61.

## Study 2b

To examine the effect of the stereotypicality of advertising on audience responses, we conducted an online experiment that manipulated stereotypicality across three levels (high, medium, and low) in a within-subject design. We measured the perceived creativity of advertisements and purchase intention toward the advertising product as a proxy for the effectiveness of advertisements as an exploratory variable.

### Participants

Using Prolific Academic, we randomly recruited participants (N = 102, women = 67, *M*_age_ = 30.69 years, *SD*_age_ = 11.45) who were residents in the UK for more than five years, based on a first-come-first-serve principle. Post-hoc power analyses using GPower suggested that we had enough power (0.96) to detect the effect of perceived stereotypicality on purchase intention at the *p* = 0.05 level (partial *η*^2^ = 0.10, effect size *f* = 0.33).

Participants consisted of White British (86%), Asian British (7%), Black British (4%), and mixed ethnicities (3%). Most participants did not report a long-standing disability (91%). They reported themselves as heterosexual (84%), LGBTQ (15%), or preferred not to say (1%). The participants gave informed consent and received £7.50 / hour.

### Materials

#### Advertisements

To manipulate the stereotypicality levels of advertising imagery, we ranked video advertisements in the advertisement pool based on perceived stereotypicality in study 2a. We selected seven advertisements per stereotypicality level: high stereotypicality, *M* = 2.20, *SD* = 0.84; medium stereotypicality, *M* = 11.23, *SD* = 1.29, and low stereotypicality, *M* = 26.11, *SD* = 5.62. Advertisements in the same ranking range (i.e., high, medium, or low) advertised products in different shopping categories (e.g., foods and sports).

#### Perceived creativity

To measure perceived creativity toward each advertisement, participants evaluated unexpectedness, uniqueness, and overall creativity on a 0–7-point Likert scale. They rated how much they agreed/disagreed with three statements: “The advertisement was typical of the kind of advertisements I see” (reversed coded), “The advertisement was unique”, and “How creative was the advertisement?”. We averaged the ratings for three statements (Cronbach’s *α* = 0.63). The higher the summed rating, the greater the perceived creativity of the advertisements.

#### Purchase intention

To measure purchase intention toward advertising products, participants rated how much they agreed/disagreed with two statements: “I am likely to purchase the product advertised” and “I would not recommend this product to a friend” (reversed coded) on a 0–7 points Likert scale. The results of scale reliability showed a low internal inconsistency of two statements (Cronbach’s *α* = 0.57), so we treated the two statements as separate variables. The higher the ratings for either statement were, the greater the willingness to purchase the advertising product for the self (purchase intention) or recommend the advertising product to a friend (recommend intention).

### Procedure

In the beginning, all participants gave consent and reported their year of residence in the UK. Only participants living in the UK for five years or more proceeded to the main task. In the main task, participants watched all advertisements with different stereotypicality levels in random order. After each advertisement, the participants were given 5 min to rate the perceived creativity of the advertisement and the purchase intention toward the advertising product. We also measured memory recall as an exploratory variable; we did not discuss these results further. The participants then provided their demographic information.

## Results

### Statistical assumptions

To prepare an appropriate dataset for repeated-measures ANOVA, we tested the relevant assumptions for this statistical test. All the variables met the assumptions of independence of variables and homogeneity of variance. There was no outlier. The skewness (between ± 1) and kurtosis (between ± 2) indicated that the data contained approximately normally distributed error. See descriptive statistics and normality tests of all measures in Supplementary Tables S10–S11 online.

### Perceived creativity

**Fig. 3:**
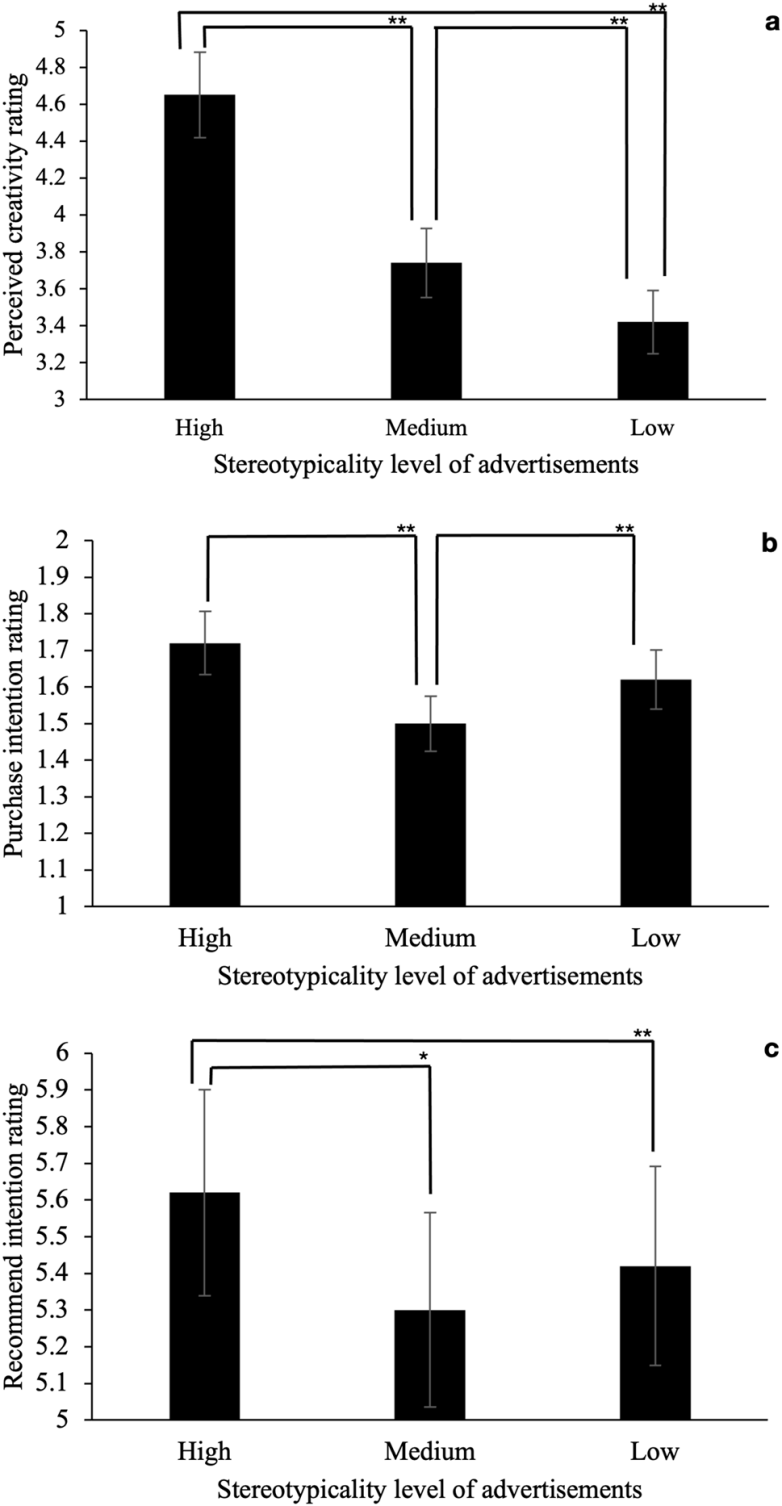
The ratings of perceived creativity toward advertisements, purchase intention toward advertising products, and recommend intention toward advertising products (with 95% confidence intervals) with different levels of stereotypicality in study 2b. The ratings that show a significant effect of stereotypicality level of advertisements is marked with one (*p* < .05) or two asterisks (*p* < .01); error bars represents ± 1 SE are shown.

We conducted a repeated-measures ANOVA to examine the effect of the stereotypicality of advertising imagery on the perceived uniqueness and creativity of the advertisements. There was a significant main effect of stereotypicality on perceived creativity, *F* (2, 100) = 148.90, *p* < 0.001, partial *η*^2^ = 0.60, *Ω* = 1.00. We followed up on this main effect with a post-hoc paired sample* t*-test. There was a significant difference between high stereotypicality and medium stereotypicality levels, *t* (101) = 13.09, *p* < 0.001, 95% CI [0.77, 1.05], such that the participants perceived advertising imagery with a high level of stereotypicality (*M* = 4.65, *SD* = 0.77) as more creative than advertising imagery with a medium level (*M* = 3.74, *SD* = 0.81). There was a significant difference between high and low stereotypicality levels, *t* (101) = 15.70, *p* < 0.001, 95% CI [1.07, 1.38], such that participants perceived advertising imagery with a high level of stereotypicality as more creative than advertising imagery with a low level (*M* = 3.42, *SD* = 0.83). There was a significant difference between the medium stereotypicality condition and the low stereotypicality condition, *t* (101) = 4.31, *p* < 0.001, 95% CI [0.17, 0.46], such that the participants perceived the advertising imagery with a medium level of stereotypicality as more creative than the advertising imagery with a low level—it was in the opposite direction than we have predicted (see Fig. 3a).

### Purchase intention

We conducted a repeated-measures ANOVA to test the effects of the stereotypicality of advertising imagery on purchase intention toward advertising products. There was a significant main effect of stereotypicality on purchase intention, *F* (2, 100) = 10.69, *p* < 0.001, partial *η*^2^ = 0.10, *Ω* = 0.33. We followed up the main effect with post-hoc pairwise comparisons. There was a significant difference between the high and medium stereotypicality conditions, *t* (101) = 4.67, *p* < 0.001, 95% CI [0.12, 0.30], such that advertising imagery with a high level of stereotypicality (*M* = 1.72, *SD* = 0.61) induced more purchase intention than did the medium level (*M* = 1.50, *SD* = 0.55). There was a significant difference between low stereotypicality condition and medium stereotypicality condition, *t* (101) = 2.76, *p* = 0.007, 95% CI [0.09, 0.20], such that advertising imagery with a low level of stereotypicality induced more purchase intention than did the medium stereotypicality condition (*M* = 1.62, *SD* = 0.56). There was no significant difference between the high stereotypicality condition and low stereotypicality condition.

This result demonstrates a sign change in the U-shaped relationship (a negative sloped and a positively sloped section in the relationship^49^) between stereotypicality and purchase intention, such that people are more willing to buy products that are marketed using high and low stereotypical imagery. We emphasize that the predictor—advertising stereotypicality—in study 2b was not continuous and may not meet the criteria for a mature U-shaped effect^49^. Therefore, we report the potential U-shaped relationship instead of a mature U-shaped effect (see Fig. 3b).

### Recommend intention

We conducted repeated-measures ANOVA to test the effects of the stereotypicality of advertising imagery on the recommended intention toward advertising products. There was a significant main effect of stereotypicality on recommend intention, *F* (2, 100) = 7.17, *p* < 0.001, partial *η*^2^ = 0.07, *Ω* = 0.27. We followed up the main effect with post-hoc pairwise comparisons. There was a significant difference between the high and medium stereotypicality conditions, *t* (101) = 3.76, *p* < 0.001, 95% CI [0.15, 0.48], such that advertising imagery with a high level of stereotypicality (*M* = 5.62, *SD* = 1.21) induced more recommend intention than did the medium level (*M* = 5.30, *SD* = 1.20). There was a significant difference between the high stereotypicality condition and the low stereotypicality condition, *t* (101) = 2.57, *p* = 0.012, 95% CI [0.05, 0.35], such that the advertising imagery with a high level of stereotypicality induced more recommend intention than did the low stereotypicality condition (*M* = 5.42, *SD* = 1.24). There was no significant difference between the medium stereotypicality and low stereotypicality conditions. These results differ from both the purchase intention and the creativity results and suggest that the relationship between purchase intention and stereotypicality is stepwise (see Fig. 3c).

### Demographic variables

We conducted an independent samples test to examine the gender differences. Results showed a significant gender difference in purchase intention in the low stereotypicality condition, *t* (101) = 2.38, *p* = 0.019, 95% CI [0.05, 0.50], such that female participants (*M* = 1.71, *SD* = 0.51) reported greater purchase intention toward the advertising imagery with a low level of stereotypicality than male participants did (*M* = 1.44, *SD* = 0.62). Female and male participants did not show significant differences in other measures. Other demographic variables did not reveal significant differences in our variables.

## Discussion

The results of study 2 support our prediction that advertising stereotypicality is associated with the audience’s perceived creativity of the advertisements and purchase intention toward the advertising products. Here, we highlight the strong correlation between perceived creativity and positive emotion in study 2a. This result supports existing research that has revealed positive emotional reactions to creativity in the media. For example, perceived creativity on Instagram helped generate positive emotions in users^50^. Creative advertisements induced more positive feelings than non-creative advertisements do^22^.

### Ethics approval

The University College London (UCL) Ethical Committee approved all studies in our research (Approval ID Number: 7453/001). We confirm that all the studies adhered to the UCL Code of Conduct for Research, UCL Research Integrity Framework, and UCL Research Data Policy. We confirm that informed consent was obtained from all participants.

## General discussion

### Theoretical implications

Across two studies, we demonstrate a relationship between stereotyping and creativity in marketing communication, which advances the understanding of corresponding psychological mechanisms. Study 1 demonstrated that our novel intervention boosted both the stereotype avoidance and originality (an indicator of creativity) of real-world marketeers, which aligns with the theory of flexibility mindsets^3^. On the one hand, the stereotype intervention probably benefited originality by breaking participants’ dominant and spontaneous associations (e.g., stereotypical associations of consumers) and encouraging divergent exploration of uncommon associations including stereotype-inconsistent labels in the labeling tasks and uncommon uses of household objects (associative mechanism). On the other hand, the increased originality might require the intentional suppression of common thoughts via controlled attention^51^. The intervention might have suppressed marketeers’ activation of stereotype-consistent information, which helped them to suppress stereotype-consistent labels and common uses of household objects (executive mechanism). Here, the executive mechanism is a more plausible explanation since increased originality via associative mechanisms would accompany increased fluency (another indicator of creativity), which was not the case in our study. Future research is needed to explore these mechanisms further.

Study 2 demonstrated that advertisements’ perceived stereotypicality and creativity are related. The results revealed a positive relationship between advertising stereotypicality and perceived creativity, such that the higher the advertising stereotypicality was, the greater the perceived creativity toward the advertisements. Perhaps our participants had been exposed to medium and low stereotypical advertising imagery more often than to high stereotypical imagery. As such, highly stereotypical advertisements might have been unexpected, driving the above association.

In addition, we found a sign change in the U-shaped relationship between advertising stereotypicality and purchase intention, such that the purchase intention toward low stereotypical advertisements was lower than those toward high stereotypical advertisements and higher than those toward medium stereotypical advertisements. Cognitive load may explain the sign change for the U-shaped effect of advertising stereotypicality on purchase intention^52^. Specifically, it may take less cognitive resources to accurately evaluate the extremes (e.g., highest and lowest) than the mediums do. Therefore, evaluating advertising imagery in medium stereotypical advertisements may cost the most cognitive resources and result in the least amount of product information processing and the lowest purchase intention. Meanwhile, processing stereotype-consistent information carries a lower cognitive load than does processing stereotype-inconsistent information. Therefore, the most stereotypical advertisements save the most cognitive load from processing product information. Given that cognitive load theory is likely the mechanism involved here, we highlight that the findings of study 2 have limited generalizability because audiences are not required to score stereotypes when they perceive advertisements in real life.

### Practical implications

Our research provides important practical implications to organizations that expend efforts and resources on boosting advertising creativity and increasing diversity, equality, and inclusion (DEI). Study 1 results suggest a similar mechanism underlies creativity and stereotyping, indicating that one may be able to 'kill two birds with one stone', provided the right intervention. Thus, training providers, organizations, and policymakers may produce and encourage more stereotype interventions based on the stereotype-creativity link.

In study 2, we are aware the possibility that the unexpectedness of high stereotypical advertisements drove the positive association between audiences’ perceived stereotypes and perceived creativity and the positive impact of high advertising stereotypicality on purchase intention. Therefore, to increase purchase intention without high stereotypical advertising, marketeers could combat advertising stereotypicality while maintain or increase advertising unexpectedness by delivering socially responsible, stereotype-inconsistent or stereotype-irrelevant advertising imagery in unexpected ways. Organizations and policymakers should be aware of the fatigue effect of overrepresenting counter-stereotype advertising images and aim to produce more novel and sustainable techniques in marketing communication to promote societal DEI. Based on previous research, we assumed that advertising stereotypes that drive social comparison may benefit purchase intention, which might be a reason that marketeers using stereotypes^33,34^. Here, our findings suggest the potential of un-stereotypical advertising imagery in serving both purchase intention and societal DEI.

### Limitations and future research

The sample size for our studies was relatively small because of the convenience sample approach employed, which may limit the statistical power of our findings. We suggest research replications that are conducted with larger samples in future studies. In addition, study 1 collected data from UK, the USA, and the Netherlands residents and study 2 mainly engaged UK residents. Therefore, our findings may not be generalizable to other cultures with different stereotypes toward social groups. We recommend research replications, especially with diverse social groups, in the future.

The self-selection sampling strategy in study 1 may have had an impact on the results interpretation. Participants who voluntarily joined the workshop were allocated to the intervention group and participants who did not volunteer were in the control group. The two groups may therefore have had different baseline levels of motivation to be less stereotypical and more creative^53^.

The CLT was designed as a real-world task for advertising, making its theoretical validity and interpretative generalizability limited. We suggest future studies to correlate stereotyping scores in the CLT with advertisement design outputs and other stereotype measurements and validate them in different contexts. In addition, study 1b measured creative thinking using AUT that captures divergent creativity only. Therefore, our results cannot be generalized to other creative thinking skills such as convergent creativity.

Moreover, future studies should be aware of participants’ inconsistent understanding of creativity. Given that one’s concept of creativity can change according to the provided information^54^, different creativity scales might affect how participants define creativity. For example, study 2a related creativity to difference, uncommonness, relevance, and meaningfulness^48^ and we found a strong positive correlation between perceived creativity and positive emotion. While study 2b related creativity with unexpectedness and uniqueness, future research may investigate whether alternative definitions of creativity produce similar results.

## Supplementary Information

Supplementary Information.

## Data Availability

The data have not been made available on a permanent third-party archive; correspondence and requests for study materials and data should be addressed to Nuoya Tan.
