# Author Correction: Investigating the relationship between stereotyping and creativity during marketing campaigns in marketeers and audiences

**DOI:** 10.1038/s41598-024-74554-7

**Published:** 2024-10-10

**Authors:** Nuoya Tan, Nimisha Parashar, Gorkan Ahmetoglu, Lasana T. Harris

**Affiliations:** 1 Present Address: Department of Management, University of Exeter, Exeter, UK; 2 Department of Experimental Psychology, University College London, London, UK; 3 Department of Clinical, Educational, and Health Psychology, University College London, London, UK; 4 Horizon Center for Doctoral Training, University of Nottingham, Nottingham, UK

Correction to: *Scientific Reports* 10.1038/s41598-024-70704-z, published online 04 September 2024

In the original version of this Article, authors Nimisha Parashar and Lasana T. Harris were incorrectly affiliated with ‘Present address: Department of Management, University of Exeter, Exeter, UK.’

Their correct affiliations are listed below:

Nimisha Parashar:

Department of Experimental Psychology, University College London, London, UK.

Horizon Center for Doctoral Training, University of Nottingham, Nottingham, UK.

Lasana T. Harris:

Department of Experimental Psychology, University College London, London, UK.

The original Article has been corrected.

